# Resettable skin interfaced microfluidic sweat collection devices with chemesthetic hydration feedback

**DOI:** 10.1038/s41467-019-13431-8

**Published:** 2019-12-04

**Authors:** Jonathan T. Reeder, Yeguang Xue, Daniel Franklin, Yujun Deng, Jungil Choi, Olivia Prado, Robin Kim, Claire Liu, Justin Hanson, John Ciraldo, Amay J. Bandodkar, Siddharth Krishnan, Alexandra Johnson, Emily Patnaude, Raudel Avila, Yonggang Huang, John A. Rogers

**Affiliations:** 1 Department of Materials Science and Engineering, McCormick School of Engineering, Northwestern University, Evanston, IL 60208 USA; 2 Center for Bio-Integrated Electronics, Northwestern University, Evanston, IL 60208 USA; 3 Department of Civil and Environmental Engineering, McCormick School of Engineering, Northwestern University, Evanston, IL 60208 USA; 4 Department of Mechanical Engineering, McCormick School of Engineering, Northwestern University, Evanston, IL 60208 USA; 5 State Key Laboratory of Mechanical System and Vibration, Shanghai Jiao Tong University, 200240 Shanghai, China; 6 School of Mechanical Engineering, Kookmin University, Seoul, 02707 Republic of Korea; 7 Department of Biomedical Engineering, McCormick School of Engineering, Northwestern University, Evanston, IL 60208 USA; 8 Department of Biology, Feinberg School of Medicine, Northwestern University, Chicago, IL 60611 USA; 9 Micro/Nano Fabrication Facility, Northwestern University, Evanston, IL 60208 USA; 10 Department of Materials Science and Engineering and Frederick Seitz Materials Research Laboratory, University of Illinois at Urbana-Champaign, Urbana, IL 61801 USA; 11 Departments of Chemistry, Electrical Engineering, Computer Science, McCormick School of Engineering, Northwestern University, Evanston, IL 60208 USA; 12 Departments of Neurological Surgery, Feinberg School of Medicine, Northwestern University, Chicago, IL 60611 USA

**Keywords:** Biomedical engineering, Mechanical engineering, Fluidics

## Abstract

Recently introduced classes of thin, soft, skin-mounted microfluidic systems offer powerful capabilities for continuous, real-time monitoring of total sweat loss, sweat rate and sweat biomarkers. Although these technologies operate without the cost, complexity, size, and weight associated with active components or power sources, rehydration events can render previous measurements irrelevant and detection of anomalous physiological events, such as high sweat loss, requires user engagement to observe colorimetric responses. Here we address these limitations through monolithic systems of pinch valves and suction pumps for purging of sweat as a reset mechanism to coincide with hydration events, microstructural optics for reversible readout of sweat loss, and effervescent pumps and chemesthetic agents for automated delivery of sensory warnings of excessive sweat loss. Human subject trials demonstrate the ability of these systems to alert users to the potential for dehydration via skin sensations initiated by sweat-triggered ejection of menthol and capsaicin.

## Abstract (web summary)

Wearables capable of collecting and analyzing sweat are of interest for athletics and health monitoring. Here, the authors report a resettable microfluidic platform comprising soft pumps and valves that provides triggered release of chemesthetic agents to alert the user of excessive sweat loss.

## Introduction

Thin, soft sensing and actuating systems that directly interface to the epidermis offer advanced capabilities in continuous health and athletic performance monitoring via readout of physical and chemical biomarkers^1^. Recent research demonstrates that in situ capture and chemical evaluation of microliter volumes of sweat, in particular, can offer promising pathways to continuous, in situ, non-invasive monitoring of biochemical processes through analysis of the rich collection of biomarkers contained in sweat^2^. Skin-mounted platforms for this purpose typically embed electrochemical and/or colorimetric sensors for precision measurement of electrolytes, metabolites, and small molecules, as diagnostics in athletic, military, and clinical care applications^3,4^. Epidermal microfluidic (epifluidic) systems for capturing, handling and locally storing sweat as it emerges from the surface of the skin offer advantages over collection techniques that use paper, hydrogel, or textile materials, due to their ability to precisely quantify local sweat loss, sweat rate, and biomarker concentration, continuously^3–8^. In their most basic forms, such devices can inform optimal hydration strategies based on accurate measurements of water and electrolyte loss, with capabilities for real-time visual feedback to the user.

Traditional techniques for determining total sweat volume and electrolyte loss rely on changes in body mass and analysis of whole body sweat^9,10^. Research by other groups establishes strong correlations between local and full-body measurements of sweat rate, sweat loss, and electrolyte (e.g. chloride) concentration and loss^11–14^. This previous work includes measurements of interpersonal variability of many body locations and highlights the high degree of correlation between local sweat loss from the forearm and full body loss. These local measurements rely on absorbent foam pads taped to the skin as the collection devices. Recent studies from our group and others demonstrate that epidermal microfluidic devices collect sweat at rates and with biomarker concentrations that are highly correlated with gold standard measurements using absorbent pads^5–8^. The findings, then, support the notion that local measurements of sweat rate/loss and electrolyte concentration can inform personalized hydration strategies, dictated by full body considerations. In this context, the assumption is that the user is in a state of euhydration (i.e. at their hydration baseline) before beginning exercise, such that measurements of loss directly relate to levels of dehydration.

Rehydration events during training or performance sessions, however, render previous measurements of sweat volume and composition obsolete. Methods for tracking sweat loss relative to a hydration baseline that accounts for such events would provide personalized and time-sensitive feedback to athletes, military personnel and to physicians in clinical care applications. The automated delivery of actionable information to the user alerting to anomalous physiological events represents an additional feature of interest in the continued development of wearable devices more generally. This broader context establishes perspectives on three capabilities that could significantly expand the utility of epifluidic devices: user-activated valves as mechanisms to control directional flow throughout the microfluidic network, integrated pumps as means for sample manipulation, extraction and mixing and responsive materials as the basis for user feedback following sweat-triggered events inside the device platform.

Control over microfluidic flows in traditional lab-on-a-chip technologies typically requires valves and/or control systems located outside of the device in setups that are incompatible with direct integration on the skin. Even integrated valves such as those formed from deformable membranes^15–17^ or from light^18^, temperature^19^ or magnetic^20^ stimuli-responsive polymers, as well as torque^21^ and electromagnetically^22,23^ actuated valves involve external equipment. By contrast, passive, directional control over flow in epifluidic devices can be achieved via choices in the dimensions of the microchannels and/or structures within them. Examples include capillary burst valves and/or superabsorbent polymer valves to enable sequential filling of discrete chambers^24–26^. Reusable, manually activated soft valves would enable enhanced, programmable fluidic operations, while retaining simplicity in design for low-cost, disposable platforms^27,28^.

The use of the sweat glands themselves as the source of pressure to drive flows in epifluidic devices is a key feature in this context. Expanding the range of capabilities to include user-controlled operations in extraction, transfer, or mixing requires mechanisms for active pumping. Microfluidic systems that combine pumps with soft valves could enable a range of important features, from the ejection of sweat as a mechanism to reset a device to an empty state, to the deployment of wearable time-sensitive assays, such as enzyme-linked immunosorbent assays (ELISA) that involve metered delivery or extraction of fluids. Electrokinetic pumps that exploit electroosmotic^29^ and electrolytic^30,31^ phenomena and optically driven pumps^32,33^ are options, but each requires complex, external control modules. The deformation of elastomeric membranes by pneumatic pressure^34^ or manual actuation^35–37^ represent attractive alternatives, but they cannot readily generate negative pressure differentials. Effervescence from the reaction of solid phase acids and bases can be used to irreversibly expel or move fluid volumes in traditional microfluidic systems^38^. Adapted methods for use in epifluidic devices would offer versatile capabilities in self-powered actuation of flow. The ability to form microfluidic pumps and valves directly integrated within a microfluidic network, using standard microfabrication processes, represents a key enabling feature for low cost, manually actuated lab-on-chip and lab-on-skin systems.

Transfer of information from a bio-integrated system to the user typically occurs via electronic or optical means. The former commonly relies on radio-frequency transmission via battery-powered Bluetooth protocols^4^ or battery-free near-field communication (NFC)^7^ technologies. Such electronic platforms can also provide electrotactile (electrical stimulation)^39,40^ and mechanical vibratory impulses^41,42^ as alerts to the user. Optical schemes include those that involve purely passive color-changing chemical reagents that respond to a biophysical or biochemical stimulus^3,43^. Such approaches offer significant advantages in simplicity and cost relative to electronic alternatives, but they require user engagement. The ability to deliver time-sensitive sensations to the skin in response to a user’s physiological status without electronics in an automated fashion would provide opportunities for alerting users to physiological anomalies.

This paper presents a set of advances in materials and microfluidic device designs that addresses these three key requirements in flow control, pumping actuation and user alerts. Specifically, the results highlight a collection of microfluidic components in soft monolithic formats, as reversible fluid indicators, reusable negative pressure pumps, chemical ejection pumps, and soft pinch valves. Combined integration of these components into systems formed via soft lithography yield epifluidic sweat devices that can be manually purged of collected sweat for resettable operation. Reversible visual indicators present local sweat loss information to the user, and a sweat-triggered chemical ejection system that includes an effervescent pump and chemesthetic agents provides sensory feedback upon certain thresholds of total sweat loss.

## Results

### Resettable epifluidic system with chemesthetic feedback

**Fig. 1:**
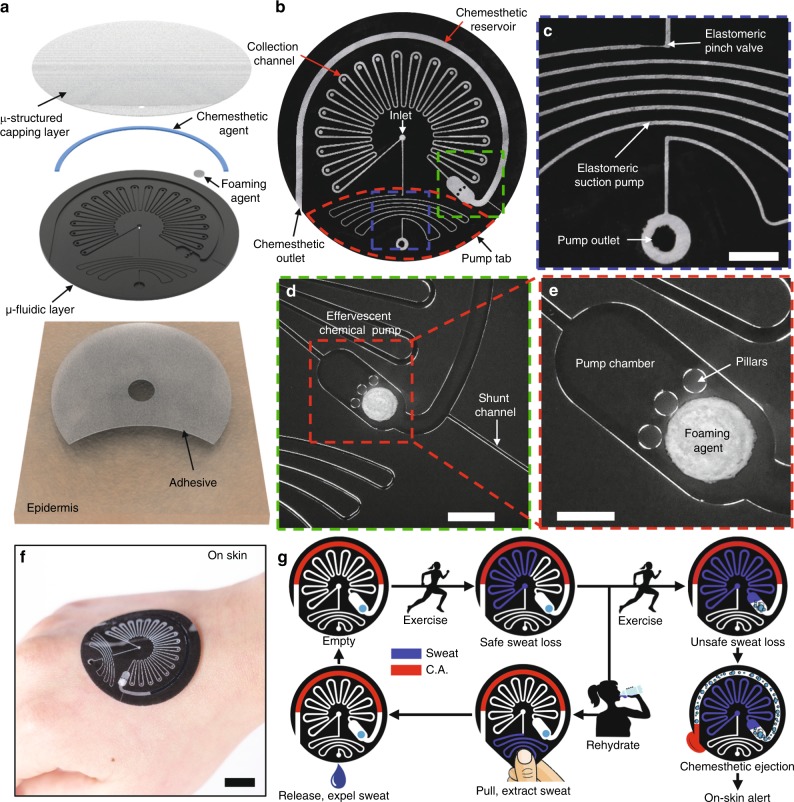
Resettable epifluidic sweat patch with chemesthetic feedback. **a** Exploded view of a resettable epifluidic sweat collection device with chemesthetic feedback. **b** The device is comprised of systems for collecting sweat, purging collected sweat, and chemesthetic ejection. **c** Collected sweat can be manually purged via an elastomeric pinch valve (ESP) and elastomeric suction pump (ESP) system. Scale bar: 2 mm. **d** At levels of filling beyond a certain volume, the sweat initiates the ejection of a chemesthetic agent for sensory feedback to the user. Optical micrograph of the effervescent pump for ejecting the chemesthetic agent. The shunt channel vents air pressure as sweat fills the device. Scale bar: 2 mm. **e** Optical micrograph of the effervescent pump, which consists of a chamber, fluid control pillars, and a water-activated foaming agent comprised of citric acid, sodium bicarbonate, and surfactant. Scale bar: 1 mm. **f** Image of the device bonded to the skin on the back of the hand. Scale bar: 1 cm. **g** Schematic flow of device operation. C.A. = chemesthetic agent.

A representative device platform that incorporates these various features appears in Fig. 1a. The system consists of a soft, microfluidic device of polydimethylsiloxane (PDMS) that conformally mounts on the epidermis to enable the capture, storage, and manual ejection of collected sweat (Fig. 1b). The device also provides sensory feedback to the user via release of a liquid chemesthetic agent onto the skin when the total sweat loss crosses a threshold. The components that support this functionality comprise a strain-actuated elastomeric suction pump (ESP) and elastomeric pinch valve (EPV) for manually expelling collected sweat (Fig. 1c), and a sweat-triggered effervescent chemical pump for ejecting a chemesthetic agent from a reservoir (Fig. 1d). In this latter system, entry of sweat into the pump chamber triggers reactions of an effervescent foaming tablet containing a solid-phase acid and base. Three pillars in this chamber serve two functions: to prevent collapse of the chamber roof during lamination of the microstructured layer and the channel layer, and to prevent premature contact between the sweat and the tablet until the chamber is filled (Fig. 1e). A dual-sided, skin-safe adhesive with an opening in the middle bonds the device to the skin and defines the sweat collection area to a small region in the center of the platform, aligned to a corresponding inlet at this location. The thin geometry and soft constituent materials enable conformal lamination of this device onto the surface of the skin, with a watertight seal that is robust against profuse sweating, deformations of the skin, and vigorous body motions (Fig. 1f).

The device in Fig. 1b supports three stages of operation as schematically illustrated in Fig. 1g. As perspiration begins, sweat enters the device from a central inlet port at the skin interface, and proceeds into a sealed collection channel (starting downward to the left, into a serpentine channel that repeats in an overall arc shape), thereby filling it by an amount that can be visualized via a reversible light-scattering effect. The EPV prevents ingress into the ESP region of the device (starting straight downward from the inlet into a serpentine channel that repeats in an overall bent horizontal shape). When the user manually grasps and pulls on the tab at the bottom of the device while covering the outlet port, a resulting suction effect pulls sweat from the collection channel into the ESP; releasing this strain ejects sweat from the outlet, thereby resetting the device. As the total volume of sweat collected into the device exceeds 25 µl, the sweat activates the effervescent chemical pump (bottom right), thereby pushing a chemical stimulant from the arc-shaped chemesthetic reservoir onto the skin to create a sensory alert to the user. The following sections describe the operation of each of the different features of the device in detail.

### Reversible visual indicators for sweat ingress

**Fig. 2:**
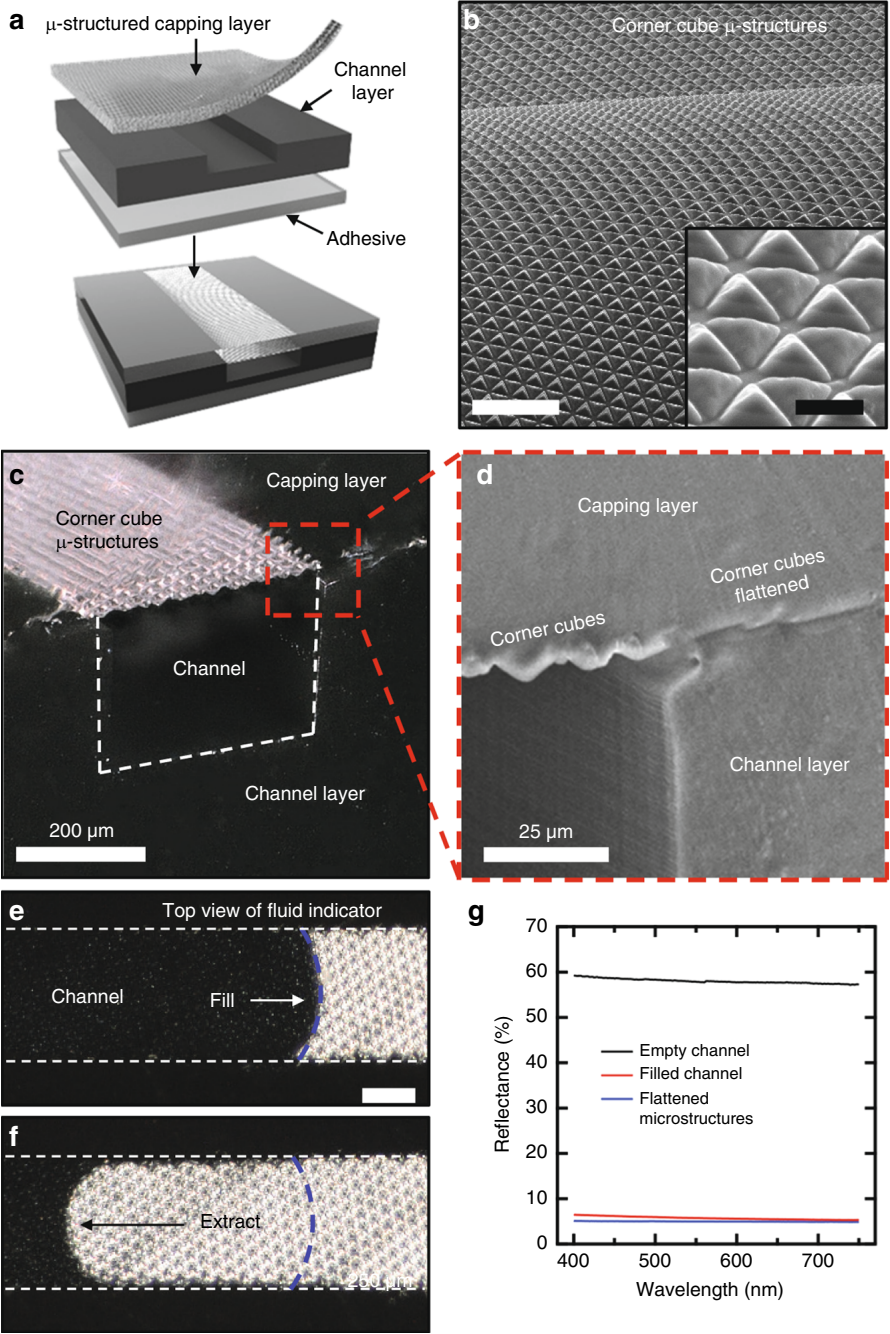
Reversible visual indicator of filling of sweat into the device. **a** Lamination of a transparent microstructured capping layer against a black base layer with molded features of relief forms microfluidic channels with embedded fluid indicators. **b** Scanning electron micrographs of microstructures formed in the geometry of reflective corner cubes on the surface of an elastomer by molding against a pattern of photoresist fabricated via grayscale lithography. Scale bar: 100 µm. Inset scale bar: 20 µm. **c** Optical micrograph of the cross section of a microfluidic channel. **d** Laminating these soft, self-adhesive, microstructures onto the channel layer flattens the relief to eliminate the associated optical effects in areas outside the channel. **e** The approximate matching of the refractive index of the sweat with the elastomer eliminates reflections from the microstructures. Scale bar: 250 µm. **f** Extraction of fluid resets these optical effects. **g** Optical reflectance of empty channels, filled channels, and flattened corner-cube microstructures. The resulting appearance changes from white, in the empty, reflective state, to black in the filled, transparent state.

Fabrication of the device involves surface treatment and lamination of a transparent capping layer of PDMS with microstructured features of surface relief onto a molded base of PDMS doped with carbon black as an opacifier, thereby forming a collection of sealed microfluidic channels and reservoirs (Fig. 2a). The contact angles of the black PDMS and microstructured PDMS are comparable to that of unmodified PDMS (Supplementary Fig. 1). The relief takes the form of an inverse replica of a grayscale patterned  photoresist (Supplementary Fig. 2) that defines reflective corner cubes (lateral dimensions of 26 µm and heights of 10–15 µm) (Fig. 2b). The relief outside of the areas of the microchannels and reservoirs flatten via mechanical collapse during the lamination process, as revealed by optical (Fig. 2c) and scanning electron (Fig. 2d) microscopy. The refractive index of PDMS (~1.4) is similar to that of sweat (~1.3), such that the filling process largely eliminates total internal reflections from these microstructures, thereby significantly modulating the optical properties (Fig. 2e) in a reversible manner (Fig. 2f). Specifically, the microstructures in empty channels diffusely reflect ~60% of incident light, to yield an appearance comparable to that of standard office paper. Sweat-filled channels reflect <10% of incident light, similar to the flattened microstructures outside the channel area. The result in this state is an appearance that is dominated by the black color of PDMS base (Fig. 2g). Cyclic purging cycles demonstrate the ability of the visual indicator to operate with negligible degradation of the optical effect for up to 20 cycles (Supplementary Fig. 3). The experiments used samples of 50 mM NaCl infused and extracted at 10 µl min^−1^ while obtaining micrographs at constant lighting conditions. For the device shown in Fig. 1b, indicator dots (*d* = 0.6 mm) that are molded in the black PDMS layer at alternating serpentine turns but disconnected from the collection channel, help elucidate the extent of filling by leaving behind a persistent optical effect after filling (1 indicator dot = 1.3 µl).

A reaction between chloride in sweat and silver chloranilate, a reagent placed near the inlet of the device, produces color changes in the sweat proportional to the chloride concentration. In this case, a green channel layer (Supplementary Fig. 4A) provides a background of a color complementary to that of the sweat upon reaction with silver chloranilate (purple) for chloride readout. This green color retains the ability to view total sweat ingress via the microstructural optics approach. Filling epifluidic devices containing the silver chloranilate reagent with 20 µl of known sodium chloride concentrations (10, 25, 50, 75, and 100 mM) yields reference color values for calibrating the chloride measurement (Supplementary Fig. 4B). Imaging with a DSLR camera with auxiliary LED lighting produces the reference images (Supplementary Fig. 4C). LAB color values can be extracted from the images after correcting the color balance using a color reference card (Supplementary Fig. 4D).

Human subject studies involved devices containing the silver chloranilate reagent mounted on the midline of both ventral forearms of subjects, followed by an indoor biking exercise for 30 min (~14 km) (Supplementary Fig. 4E). Reference sweat samples were manually collected directly from the skin in centrifuge tubes. Imaging of devices using a DSLR camera enabled extraction of the LAB color values in post processing after correcting for color balance using a color reference card. Converting the a* and b* color values using the calibration curve produced the chloride concentration measurement. Reference chloride measurements were performed on a commercial chloridometer with a coefficient of variation of 1.02% at 100 mM. The results show excellent correlations between values measured via colorimetric readout and via the chloridometer (Supplementary Fig. 4F).

Sweat electrolyte levels remain relatively constant at constant exercise intensity^44,45^. As a result, a single measurement of chloride at the beginning of a session can help to inform users of their hydration needs. The ejection of the colorimetric reagent during the first purging cycle will enable the device to retain function as a sweat loss monitor in the manner previously described. The ability to deliver accurate and timely information to a user about levels of sweat loss and electrolyte loss represents an important advance in personalized hydration feedback.

### Soft, strain-actuated microfluidic valves

**Fig. 3:**
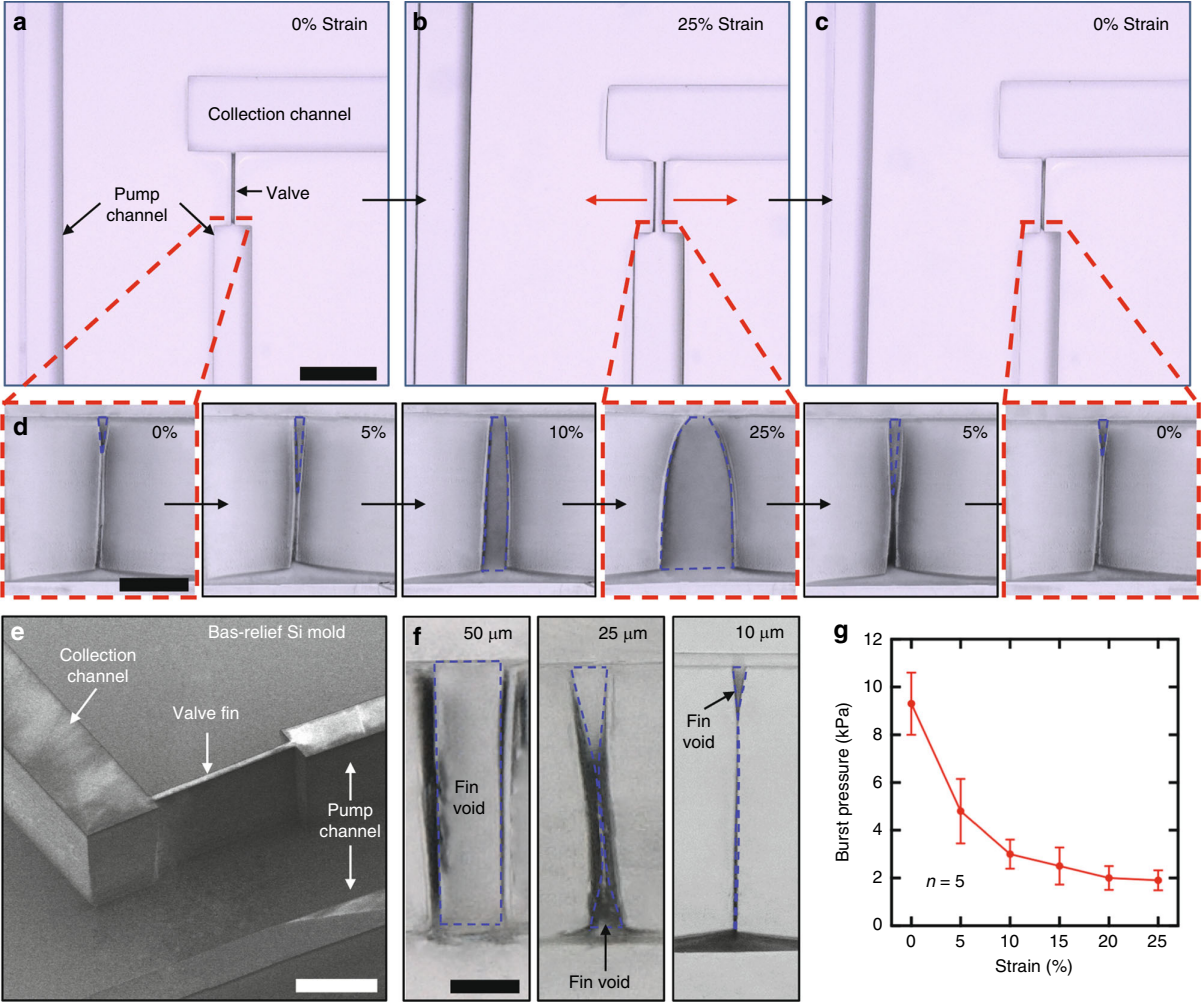
Elastomeric, strain-actuated pinch valve. The mechanically actuated opening and reversible collapse of high-aspect ratio voids in molded elastomers form the basis of strain-actuated pinch valves. **a** Top view of a soft, microfluidic pinch valve shortly after lamination with the capping layer to complete the assembly of the device structure. Scale bar: 500 µm. **b** Strain applied perpendicular to the valve overcomes van der Waal forces between the sidewalls of the elastomer to open the valve. **c** Relaxing the strain reforms of the adhesive bond between the walls. **d** Cross-sectional micrographs of a valve with an as-fabricated width of 10 µm and height of 250 µm for strains between 0% and 25%. Scale bar: 100 µm. **e** Scanning electron micrograph of the corresponding silicon mold, illustrating the fin structure used to define the valve. Scale bar: 250 µm. **f** Cross-sectional micrographs of PDMS valve sidewalls formed from silicon fins with widths of 50 µm (left), 25 µm (middle), and 10 µm (right). Scale bar: 50 µm. **g** Burst pressure of valves formed from 10 µm wide fins for strains of up to 25%. The error bars indicate standard deviation.

The microfluidic channel leading from the skin bifurcates immediately after the inlet. The normally closed EPV prevents sweat ingress into the channel that leads to the ESP (starting straight downward in Fig. 1b), to ensure that sweat flows entirely into the serpentine collection channel (starting downward to the left in Fig. 1b). In the EPV structure, van der Waals forces provide temporary, reversible adhesion between the surfaces of two parallel features of PDMS formed during the channel molding process (Fig. 3a). Lateral strain applied in a direction perpendicular to the sidewalls overcomes these adhesive forces to increase the cross-sectional area. This deformation forms the basis of a valve (Fig. 3b). Releasing this strain re-forms the adhesive bond between the PDMS features, thereby sealing the valve and returning it to its normally closed configuration (Fig. 3c).

Cross-sectional micrographs illustrate the propagation of the crack that increases the cross-sectional area from ~225 to ~17,900 µm^2^ as the strain increases to 25%, along with the subsequent closing of this crack upon strain relaxation (Fig. 3d). An important feature of the operation is that complete, intimate closure of the valve is unnecessary because the capillary burst pressure associated even with a partially open valve can exceed that associated with the sweat glands (<6 kPa), modulated by sources of additional pressure that might arise from motion-induced inertial effects or strains from deformation of the skin during activity. Fabrication of the EPV relies on a high-aspect ratio (25:1) silicon fin formed in bas-relief on the corresponding mold (Fig. 3e). The result creates voids in PDMS that collapse and self-adhere following demolding. The mechanics of collapse depends strongly on the width of the fin, as is illustrated with valves formed from fins with widths of 50, 25, and 10 µm (Fig. 3f). Measurements of the burst pressure of a valve formed with a 10 µm feature as a function of applied strain up to 25% reveal the key behaviors and the ability to block flows with pressures up to 10 kPa (Fig. 3g). A 50 mM NaCl solution was injected into the collection channel of a device with a blocked outlet at increasing pressure levels until flow was observed in the pump channel. The collection and pump channels were then manually purged via straining of the pump tab to 25% while the collection channel outlet was open. The hydrophobic nature of PDMS largely prevents residual water from collecting in the channel and valve after purging (Supplementary Fig. 5A). The process was repeated 20 times, after which three cycles were performed with the pump tab held at 25% strain for 1 min. This process was repeated for three devices. The results indicate no degradation of burst pressure during the cyclic tests (Supplementary Fig. 5B). Additionally, the morphology of a valve that underwent the identical cyclic straining process exhibited no observable morphological changes (Supplementary Fig. 5C). These studies illustrate the repeatability of EPVs after multiple straining cycles and resistance to degradation of the valve morphology. The geometry of the valve test structure is shown in Supplementary Fig. 6A.

### Soft, strain-actuated microfluidic suction pumps

**Fig. 4:**
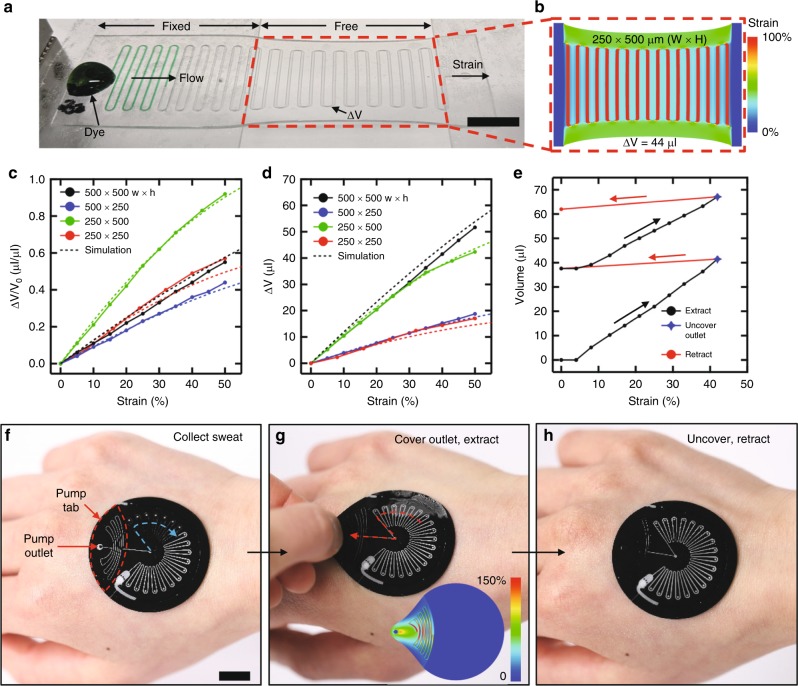
Elastomeric, strain-actuated microfluidic purge system. **a** Demonstration of strain-induced suction in a serpentine microchannel embedded in PDMS to induce flow of a liquid dye (Δ*V* = change in volume). Scale bar: 1 cm. **b** 3D finite-element analysis of embedded microchannels with a cross section of 500 × 250 µm after straining to 50% indicates a capability to extract 44 µl of liquid. **c** Volume extraction as a percentage of initial pump volume and dependence on cross-sectional dimensions. **d** Total volume extracted and dependence on cross-sectional dimensions. **e** Volume extracted from the collection channel during multi-cycles straining of a resettable device. Covering the outlet hole and applying lateral strain to the pull tab extracts sweat. Uncovering the outlet and subsequently releasing the strain purges the sweat from the device via the outlet. **f** Collected sweat in a resettable epifluidic device. Scale bar: 1 cm. **g** Covering the outlet and extending the pull tab extracts sweat into the pump channel. Inset: Strain distribution during 100% strain of the pull tab (7.75 mm lateral deformation). **h** Uncovering the pump outlet and releasing the strain purges the extracted sweat via the pump outlet.

The ESP relies on serpentine microchannels that form a variable-volume cavity to generate a negative pressure differential upon lateral extension of the serpentine via strain applied to the tab, as visualized via the extraction of fluid from an aperture at the end of the microchannel (Fig. 4a). The negative pressure extracts fluid in a manner governed by the magnitude of the applied strain and the geometric dimensions of the pump. The ability to extract fluid, as opposed to expel, represents a key aspect of this work as it enables collected sweat to be purged without affecting the effervescent pump and chemesthetic agent, and minimizes the opportunity for inadvertent actuation of the pump during use. Experiments and 3D modeling results illustrate the influence of the size and aspect ratio of the embedded microchannels on the volume extraction (Supplementary Fig. 7). Structures with high aspect ratios (2:1 height:width) generate a change in volume of 44 µl at 100% strain (Fig. 4b) as shown by finite-element analysis. Experimental and computational results for changes in the volume of strained serpentine microchannels of various cross sections (250 × 250, 500 × 500, 250 × 500, 500 × 250) after normalizing to the initial pump volume are illustrated in Fig. 4c. The differences in the magnitude of volume change are shown in Fig. 4d. The results indicate that the extraction volume depends more strongly on the height of the microchannels than on the width, as confirmed by finite-element analysis. The geometries of the pump test structures are shown in Fig. S6B.

A complete system for purging sweat from the device consists of an ESP, EPV, and a momentary valve formed by manually occluding the outlet of the ESP, as illustrated in Supplementary Fig. 6C. These components work together to enable extraction of sweat via a manual, two-stroke lateral deformation and subsequent retraction of a pull tab. The EPV prevents sweat flow into the purge pump during sweat ingress. Gripping the ESP outlet and applying lateral strain simultaneously forms an air-tight seal between the pump channel and external environment via the users thumb, opens the EPV, and generates suction to extract sweat into the ESP. Subsequent release of the ESP outlet followed by retraction of the pump tab pushes the extracted fluid in the pump channel out through the outlet. Minimal backflow of extracted sweat into the collection channel occurs during retraction due to the relatively small aperture of the valve (~225 µm when closed) as compared to the pump outlet (196,000 µm^2^, hole with *r* = 250 µm) (Fig. 4e). Designing the ESP channel to form concentric arcs about the point of strain application (the pump outlet) maximizes the strain that is manifested parallel to the channel cross section (Supplementary Fig. 8). The absence of skin adhesive under the tab region of the device allows the manual manipulations and deformations needed to operate the EPV and ESP while adhered to the back of the hand (Fig. 4f–h). Direct observation of the collection channel during purging allows the user to initiate additional purge cycles as needed, obviating the need for precise actuation of the ESP.

### Sweat-triggered chemesthetic alerts

**Fig. 5:**
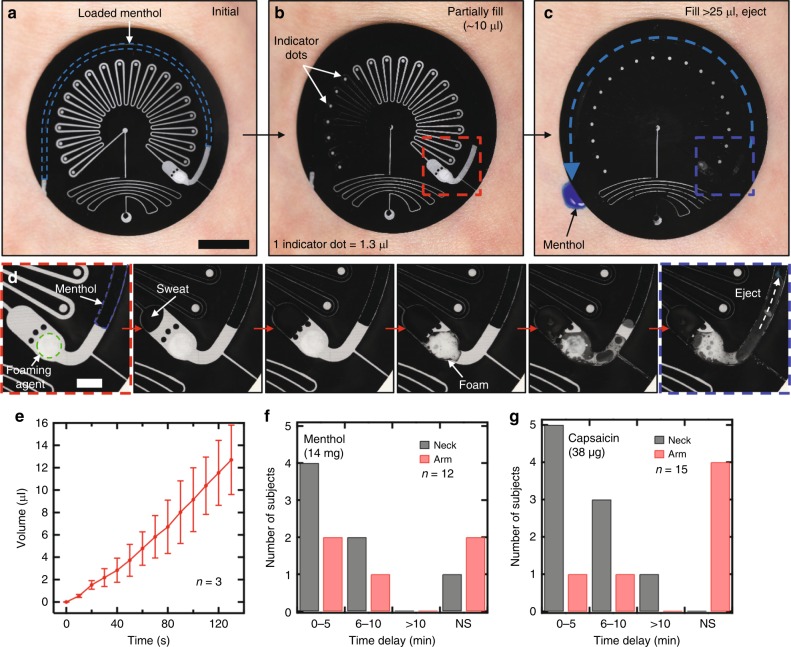
Sweat-triggered chemesthetic feedback. **a** A liquid menthol solution resides in a semicircular channel that defines the chemesthetic reservoir. Scale bar: 1 cm. **b** Sweat enters into the collection channel as air pressure is relieved through the shunt channel. **c** The contact of sweat with the citric acid/sodium bicarbonate foaming agent produces CO_2_ which ejects the menthol. **d** After filling the collection channel, sweat enters into a reaction chamber. As the sweat fills beyond a set of containment pillars in this chamber it encounters a foaming agent tablet to initiate a reaction that forms CO_2_ gas, thereby producing pressure that ejects the liquid menthol. The small cross section of the shunt channel (25 × 250 µm) prevents significant flow through the shunt. Scale bar: 1 cm. **e** Rate of ejection of a menthol solution. The error bars indicate standard deviation. **f** Ejection of 14 mg of menthol solution elicits a sensation within 10 min of ejecting for 9/12 subjects. **g** Ejection of 38 µg of capsaicin elicits a response within 10 min of ejecting for 12/16 subjects. NS = no sensation.

Solutions of menthol or capsaicin serve as the chemesthetic agents, preloaded into an associated reservoir in the form of a semi-circular channel that lies around the perimeter of the device (Fig. 5a). The device can collect sweat, with resettable capabilities described in the previous section, for total sweat volumes <25 µl (Fig. 5b). For volumes >25 µl, the sweat automatically triggers the ejection of the chemesthetic solution to create a physical sensation as an alert to the user (Fig. 5c). Ejection of the chemesthetic agent proceeds in an irreversible fashion. The volume of the collection channel, 25 µl in this case, represents the volume collected from a 6 mm diameter collection area on the volar forearm concomitant with an ~1% change in body mass during exercise after correcting for fluid intake, assuming that secondary losses, such as due to expectoration and respiration are negligible^5,6^. A body mass loss of 1% represents the accepted threshold for thirst sensation and for impaired thermoregulation^9,46^.

As in Fig. 5d, the pump that drives this ejection process relies on sweat-initiated reactions associated with an effervescent tablet (1 mg) comprised of a solid-phase acid, base, and liquid surfactant loaded into a pump chamber. The outlet of the pump chamber bifurcates and leads to both the chemesthetic reservoir and a shunt channel, the latter of which serves to relieve backpressure during sweat collection. Chemesthetic ejection proceeds as follows: sweat enters the effervescent pump chamber (volume = 1.5 µl), a trio of molded PDMS pillars mitigate inertial effects that could lead to inadvertent contact of the sweat with the effervescent tablet before the chamber is full, sweat proceeds past the pillars and reacts with the tablet to produce CO_2_ and a foam, stabilized by the surfactant, that expels the chemesthetic agent (Supplementary Fig. 9). Fluid flow through the shunt is minimal due to the small cross-sectional area of this channel (25 × 250 µm) relative to the chemesthetic reservoir (1000 × 250 µm). Ejection of 15 µl of chemesthetic solution occurs within ~120 s (Fig. 5e), to create well-defined skin sensations.

On-body trials during exercise reveal the chemesthetic sensitivity associated with two mounting locations for menthol (14 mg) (Fig. 5f) and capsaicin (38 µg) (Fig. 5g). Doses based on concentrations of commercial topical medications containing menthol and capsaicin (retrieved from the FDA National Drug Code Directory), and protocols approval by an Institutional Review Board, ensure the safety of participants. The evaluations involve epifluidic devices on the volar forearm and side of the neck during indoor biking until ejection of the chemesthetic agent, without an ability to visually observe the devices (Supplementary Fig. 10). The results indicate that the doses lie above the lower limen (i.e. sensation threshold) for 9/12 menthol subjects and 12/16 of capsaicin subjects, with all sensations registered within 10 min of ejection. Ejection of menthol on the neck and forearm results in sensations in 6/7 and 3/5 subjects, respectively. For capsaicin, the corresponding results are 9/9 and 2/6. In general, ejections on the neck lead to stronger sensations (15/18 subjects) than those on the forearm (5/11 subjects), consistent with previous findings of the regional differences in cutaneous sensitivity of menthol and capsaicin^47^. Particularly strong sensations occur for ejection of capsaicin on the neck. The delay between ejection of the chemesthetic agent and the emergence of skin sensations shown in Fig. 5f, g is dictated in part by inter/intrapersonal variances. These variances in sensory reactions to chemesthetic irritants is generally accepted in the literature^47^.

The ability for ejection of the chemesthetic agent to alert the user to high levels of sweat loss in a timely manner relies on both the sweat and skin sensitivity of the user. Sweat loss can vary substantially across the body based on intraindividual and interindividual factors, such as activity, environment, and physique. Tennis players have one of the highest sweat rates of athletes in mainstream sports, with the average cramp-prone male player losing 2.6 L h^−1^ ^10^. At this rate, a 90 kg player will lose ~430 ml within 10 min, or ~0.48% of their body mass. Emergence of the chemesthetic sensation within 10 min of reaching 1% body mass loss will result in the user receiving the alert when they near 1.48% body mass loss, well before they reach the threshold of dehydration (2% body mass loss). Expanded studies will help to establish the efficacy of chemesthetic skin sensations to alert users during rigorous exercise, as well as the dependence of sensory limen on environmental and interpersonal variance.

**Fig. 6:**
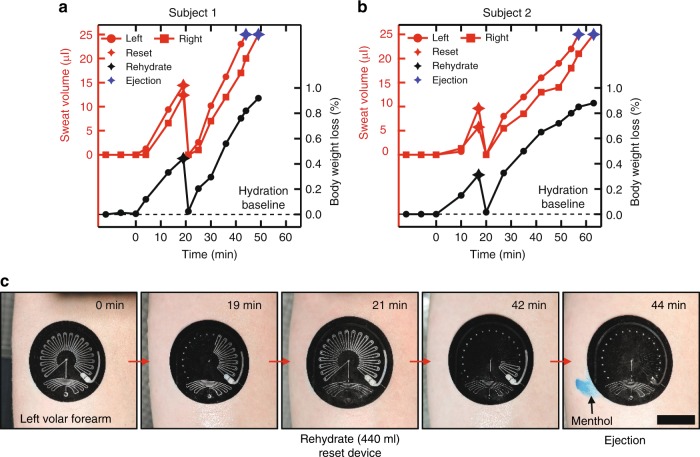
Resettable sweat collection and chemesthetic ejection. Data from sauna trials for two subjects demonstrate the ability of these systems to operate in real world environments. Good correlation between epifluidic sweat collection and body weight loss is observed before and after resetting and rehydration for **a** Subject 1 and **b** Subject 2. **c** Representative images from the trial with Subject 1. Scale bar: 2 cm.

Figure 6 demonstrates the ability of these systems (microstructured optical visual readout, pinch valves and suction pumps, and effervescent pumps and chemesthetic agents) to operate in on-body trials. Two subjects wore devices on both volar forearms during exposure to thermal stress in a sauna. Regular measurements of body mass loss and local sweat loss establish a robust correlation between local sweat volume measurements and body mass loss (Fig. 6a, b). Imbibing a volume of water corresponding to the subject’s total body mass loss returned the subjects to their hydration baselines and was performed coincident with the purging of collected sweat via the ESP. Subsequent thermal exposure without rehydrating for an additional 30 and 40 min for Subjects 1 and 2, respectively, prompted fluid losses of ~1% body mass and 25 µl of local sweat loss. Images of a device on Subject 1 illustrate the ability of the device to capture and display total sweat volume in a manner consistent with changes in body mass loss, be manually reset by the user after rehydration via the ESP, and initiate ejection of the chemesthetic agent after exceeding 25 µl of local sweat loss.

Effects of sweat gland occlusion via the adhesive layer can be virtually eliminated via the use of structured adhesives designed to allow release and flow of sweat laterally to the perimeter of the devices. Studies to demonstrate the effects involve subjects wearing (a) epifluidic devices with adhesives patterned into geometries shown schematically in Supplementary Fig. 11A and (b) absorbent pads positioned adjacent to these devices and held to the skin with Tegaderm adhesives, both on the inner volar forearms during indoor biking trials (Supplementary Fig. 11B). The ingress of sweat into the epifluidic devices after 30 min of indoor biking recorded via a smartphone camera and the corresponding change in mass of the absorbent pads yield estimates of the areal sweat loss. The results obtained with the epifluidic device are the same, to within experimental uncertainties, as those with the absorbent pad. These findings indicate the ability to eliminate effects of occluded sweat glands with a simple modification to the device, i.e. use of structured adhesives (Supplementary Fig. 11C). Previous work has demonstrated similar results for patterned adhesive layers^5,6^.

## Discussion

This paper establishes a set of soft microfluidic components and systems for sweat capture, collection, and electrolyte analysis, with capabilities for automated feedback to the user, all without electronics or active components. Fabrication of manually operated, monolithic, integrated microfluidic systems, using standard microfabrication workflows, represents a key enabling feature for low cost, manually actuated lab-on-chip and lab-on-skin systems. The essential elements include reversible, visual indicators of fluid ingress, ESPs and EPVs, and chemical ejection pumps, all in compliant, thin formats that establish water-tight, comfortable interfaces to the skin. Systematic benchtop testing demonstrates the cyclic stability of EPVs and microstructural optics to operate without degradation in the presence of artificial sweat for up to 20 purge cycles. Detailed modeling of the valves and pumps reveal the dependence of their operation on geometrical and materials parameters. The results significantly expand on capabilities in epifluidic systems, to enable user-initiated purging of sweat from the system to reset the operation, and sweat-triggered release of chemesthetic agents to produce sensory alerts to possible dehydration, all in a passive manner with minimal complexity and cost. Human subject trials demonstrate the ability to track fluid loss while accounting for rehydration events, and to exploit menthol and capsaicin as chemesthetic agents. Chloride measurements via colorized substrates and microstructural optics provide colorimetric readout of electrolyte loss. Expanded studies will serve to verify the utility of local sweat measurements and chemesthetic alerts generated by these classes of devices to provide personalized hydration feedback. More generally, the classes of microfluidic components introduced here could support additional levels of functionality, such as manual time sampling for external chemical analysis and time-sensitive mixing with chemical assay reagents.

## Methods

### Fabrication of the channel layer mold

Exposing a 10 μm-thick layer of negative photoresist (KMPR 1010; spin cast at 3000 RPM and 1000 RPM s^−1^ for 30 s and soft baked at 110 °C for 5 min) on a silicon wafer to ultraviolet light (420 mJ cm^−2^) through a chrome mask followed by soft baking (110 °C for 5 min) and developing (AZ 917MIF, 3 min) produced patterns in the resist. A deep reactive ion etch (DRIE) Bosch process created trenches in the exposed regions of the silicon wafer to a depth to 250 μm (STS Pegasus ICP-DRIE, SPTS Technologies Ltd.). Ending the Bosch process on a passivation step left a conformal fluorocarbon layer on the silicon to facilitate release during the production of replicas in PDMS via soft lithographic molding.

### Fabrication of the microstructured layer mold

Grayscale exposure (Heidelberg MLA150) of a 15 µm-thick layer of positive photoresist (AZ 4620 spin cast at 1000 RPM, 500 RPM s^−1^ for 40 s and soft baked at 115 °C for 180 s) on a silicon wafer-defined patterns in the geometry of pyramidal corner cubes. Development in AZ 400K diluted at 1:4 with DI water for 5 min formed the final structures. Soft lithographic molding defined an inverse copy of the pattern of photoresist in PDMS (10:1 base to curing agent). The PDMS structures enabled formation of replicas of the original photoresist pattern in a UV-cured optical epoxy (Norland Optical Adhesive 81). These replicas were used as molds to produce microstructured capping layers in PDMS.

### Fabrication of silicone microfluidic systems

Spin coating a layer of PDMS (20:1 base to curing agent, mixed in a planetary mixer (Thinky ARE-310) for 30 s at 2000 RPM) on molds for the channels (200 RPM, 200 RPM s^−1^, 30 s) and the microstructures (400 RPM, 200 RPM s^−1^, 30 s) followed by partial curing for 1.5 h at 80 °C yielded solid replicas with tacky surfaces. The addition of 3 wt% each of green and white silicone pigments (Silc Pig, Smooth-on) forms a green backdrop for devices configured to allow measurements of chloride concentration. Corona treatment (ElectroTechnic BD-20AC) of both layers followed by lamination with applied pressure collapsed and bonded the microstructured surfaces in regions outside the channel areas. The use of partially cured PDMS, surface activation, and firm pressure applied by hand within 5 min of surface activation ensures complete bonding. Baking for 6 h at 80 °C completed the curing process and ensured strong interfacial bonding. A CO_2_ laser cutting system (Universal Laser, VLS3.50) formed the necessary patterns in a uniform sheet of skin adhesive (3M 1524). Corona treatment of the backside of the device followed by lamination to the adhesive yielded a strong bond. A circular steel punch (*d* = 40 mm) defined the outer diameter of the completed system.

### Measurements of contact angle

A VCA Optima XE contact angle measurement system defined the contact angle associated with 0.5 μl droplets of DI water.

### Characterization of strain-dependent burst pressure of EPVs

Mounting valve test structures to a Plexiglas holder with the device inlet aligned to a 2 mm aperture in the holder prepared the device for filling. A pressure-driven flow controller (Fluigent MFCS-EZ) pumped dyed water at controlled pressures through the aperture and into the device, with test geometry shown in Supplementary Fig. 3A. Visual inspection of the device during a slow, monotonic increase in the pressure with increments of 0.5 kPa and hold times of 30 s revealed the burst pressure of the valve. Lateral strains applied by a controlled stretching stage (Aerotech ATS100) in 5% strain increments at a rate of 1 mm s^−1^ deformed the test structure.

### Suction pump characterization

Aluminum (6061) structures machined using a three-axis mill (Roland MDX-540) served as the molds for the suction pump test structures (Supplementary Fig. 3B). Vacuum desiccation for 30 min removed bubbles from PDMS cast in these molds. Curing occurred on a hot plate at 80 °C for 5 h. A steel punch formed the inlet hole (1 mm). Corona treating the molded layer and the flat layer followed by lamination with applied pressure and baking at 80 °C for 12 h completed the bonding process. Mounting the ends of the sample to Plexiglas holders with an adhesive layer (3M 1524) prepared the device for testing. Placing a droplet of dyed water on the outlet and leaving the inlet sealed enabled visualization of changes the volume of the strained portion of the pump by tracking the front of fluid ingress. Lateral deformation applied by a controlled stretching stage (Aerotech ATS100) in 1.25 mm increments (corresponding to increments of 5% strain) at a rate of 1 mm s^−1^ induced volume changes in the embedded microchannel. Imaging with a smartphone camera (Google Pixel 3) provided a visual record of changes in the front of fluid ingress, subsequently converted to extracted volume based on the channel geometry.

### Finite-element analysis of the suction pump

Three-dimensional (3D) FEA was used to study the performance of the ESP with different geometries. Eight-node 3D solid elements (Abaqus element type C3D8R) were used for the microfluidic channels. The Young’s modulus of the microfluidic channel cover/wall material used in FEA was 46 kPa. Pseudoelements with extremely low modulus (<1/1000 of microfluidic channel cover/wall material modulus) were filled in the microfluidic channels for calculation of inner volume change without interfering with the deformation of the channel. Left and right boundaries of the suction pump region are clamped during stretching in FEA, mimicking the experimental setup shown in Fig. 4a. 3D FEA was also used to study the deformation and calculate volume change in the microfluidic channel of the pump tab in the real device. The boundary conditions applied in FEA were also consistent with the process of manually pulling the tab.

### Optical measurements

A UV/VIS spectrophotometer with integrating sphere module (LAMBDA 1050, Perkin Elmer) enabled diffuse reflectance measurements of empty microchannels, water-filled microchannels, and flattened microstructures on black PDMS. Measurements were taken with reference to a diffuse white reflectance standard (Spectralon, Labsphere).

### Chemesthetic ejection system

Menthol (Acros Organics) and capsaicin (Sigma Aldrich) were dissolved in propylene glycol (Sigma Aldrich) at 90 and 0.25 wt%, respectively. The addition of 2 wt% polyvinylpyrrolidone (Sigma Aldrich) increased the viscosity of both solutions to prevent inadvertent ejection due to inertial effects associated with motion during activities. Addition of 1% of an oil-based food dye (Winton Candy Colors) colored the menthol and capsaicin solutions blue and red, respectively. Injection of 15 µl of an individual chemesthetic solution into the epifluidic device via the outlet filled the chemesthetic channel, corresponding to 14 mg menthol and 38 µg capsaicin. Stoichiometric quantities of citric acid and sodium bicarbonate (1.2:1 sodium bicarbonate:citric acid by weight) were mixed with an additional 3 wt% polysorbate 20. The fine particle sizes of the dry components (<50 µm) facilitated rapid dissolution and gas production. Manual tamping of ~1 mg of the acid/base/surfactant mixture in a cylindrical aluminum mold formed a 200 µm tall, 2 mm diameter dry effervescent tablet. Placing the effervescent tablet in the pump chamber prior to lamination of the capping layer prepared the chemesthetic pump.

### Chemesthetic on-body trials

The experimental protocols for the on-body chemesthetic studies were approved by the Institutional Review Board of Northwestern University (STU00207078), and all subjects gave written informed consent before participation. Epifluidic test structures filled with a menthol or capsaicin solution were placed on the volar forearm and side of the neck. Participants rode a stationary bike until ejection of the chemesthetic agent (~30 min of exercise), at which point a study administrator took note of the ejection time. The particulates were blinded from observing the devices and were unaware of the ejection time. Participants were asked to indicate the time of first sensation and the results were noted by the study administrator. Mounting locations were cleaned with an alcohol pad and lidocaine cream was applied to the site of the capsaicin device to minimize discomfort after completion of the trial.

### Resetting and chemesthetic ejection trials

Imaging devices used sweat volume trials with a smart phone (Google Pixel 3) recorded the device status periodically during exposure to thermal stress. Collected sweat volume was determined by counting the number of completed serpentines to the nearest half serpentine (±0.75 ml). Measurements of percentage of body weight loss (total body loss) by a body weight scale with 4-g precision (Adams GFK 330ah) enabled high-precision measurements of percentage of body weight loss. The animal (dynamic) measurement mode was used, which filters variations resulting from movement during the weighing procedure.

### Chloride measurements

Filling epifluidic devices containing the silver chloranilate reagent with 20 ml of known sodium chloride concentrations (10, 25, 50, 75, and 100 mM) produced the reference color values for calibrating the chloride measurement. Imaging of the resulting product using a Canon EOS Rebel T6i DSLR camera with auxiliary LED lighting yielded the reference images. LAB color values were extracted from the images after correcting the color balance using a 24-color card (Camera Trax).

Devices containing the silver chloranilate reagent were mounted on the midline of the ventral forearm. Subjects biked for 30 min (~14 km). Reference sweat samples were manually collected in centrifuge tubes. Imaging of devices using a Canon EOS Rebel T6i DSLR camera enabled extraction of the LAB color values in Photoshop after correcting for color balance using a 24-color card. Converting the a* and b* color values using the calibration curve produced the chloride concentration measurement. Reference chloride measurements were performed on a Chloro-Chek Chloridometer with a coefficient of variation of 1.02% at 100 mM.

### Reporting summary

Further information on research design is available in the Nature Research Reporting Summary linked to this article.

## Supplementary information

Supplementary Information

Reporting Summary

## Data Availability

The data that support the findings of this study are available from the corresponding author on reasonable request.
