# Supplementary material for: Resettable skin interfaced microfluidic sweat collection devices with chemesthetic hydration feedback
Source: Nat Commun. 2019 Dec 4;10:5513. doi: 10.1038/s41467-019-13431-8 (PMC6892844; doi:10.1038/s41467-019-13431-8)
Supplement: Supplementary file 1 — Supplementary Information [file 41467_2019_13431_MOESM1_ESM.pdf]

Supplementary Information

Resettable Skin Interfaced Microfluidic Sweat Collection Devices with Chemesthetic Hydration Feedback

Jonathan T. Reeder[1,2], Yeguang Xue[3,4], Daniel Franklin[2], Yujun Deng[3,4,5], Jungil Choi[1,2,6], Olivia Prado[7], Robin Kim[7], Claire Liu[7], Justin Hanson[8], John Ciraldo[9], Amay J. Bandodkar[1,2], Siddharth Krishnan[10], Alexandra Johnson[6], Emily Patnaude[6], Raudel Avila[3,4], Yonggang Huang[1,3,4], John A. Rogers[1,2,4,5,11,12]*

[1]Department of Materials Science and Engineering, McCormick School of Engineering, Northwestern University, Evanston, IL 60208, USA

[2]Center for Bio-Integrated Electronics, Northwestern University, Evanston, IL 60208, USA

[3]Department of Civil and Environmental Engineering, McCormick School of Engineering, Northwestern University, Evanston, IL 60208, USA

[4]Department of Mechanical Engineering, McCormick School of Engineering, Northwestern University, Evanston, IL 60208, USA

[5]State Key Laboratory of Mechanical System and Vibration, Shanghai Jiao Tong University, Shanghai, 200240, China

[6]School of Mechanical Engineering, Kookmin University, Seoul 02707, Republic of Korea

[7]Department of Biomedical Engineering, McCormick School of Engineering, Northwestern University, Evanston, IL 60208, USA

[8]Department of Biology, Feinberg School of Medicine, Northwestern University, Chicago IL 60611 USA

[9]Micro/Nano Fabrication Facility, Northwestern University, Evanston, IL 60208, USA

[10]Department of Materials Science and Engineering and Frederick Seitz Materials Research Laboratory, University of Illinois at Urbana-Champaign, Urbana, IL 61801, USA

[11]Departments of Chemistry, Electrical Engineering, Computer Science, McCormick School of Engineering, Northwestern University, Evanston, IL 60208, USA

[12]Departments of Neurological Surgery, Feinberg School of Medicine, Northwestern University, Chicago IL 60611 USA

Corresponding author: jrogers@northwestern.edu

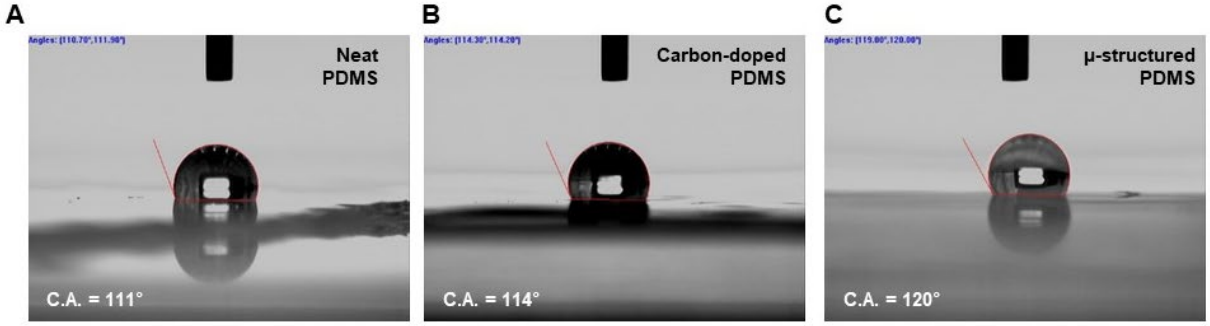

Supplementary Figure 1. Contact angle of PDMS surfaces. A) Neat PDMS. B) PDMS doped with carbon black. C) Microstructured PDMS.

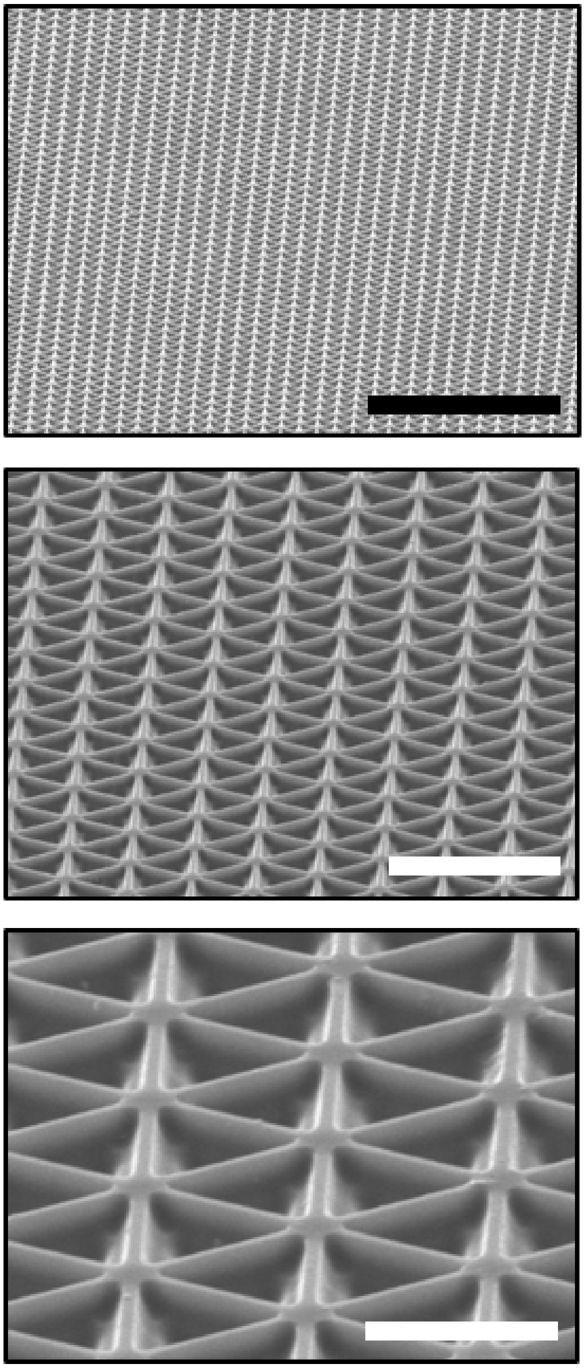

Supplementary Figure 2. Fluid indicator molds. Scanning electron microscopy of epoxy molds fabricated from a master photoresist mold formed via grayscale lithography. Scale bars: 200, 50, 20 µm (top to bottom)

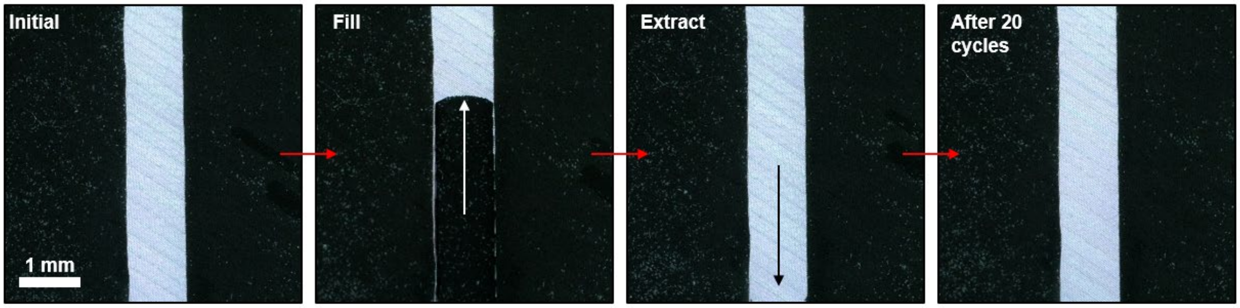

Supplementary Figure 3. **Microstructural optics repeatability.** Effect of cyclic filling and extraction of 50 mM NaCl. Scale bar: 1 mm.

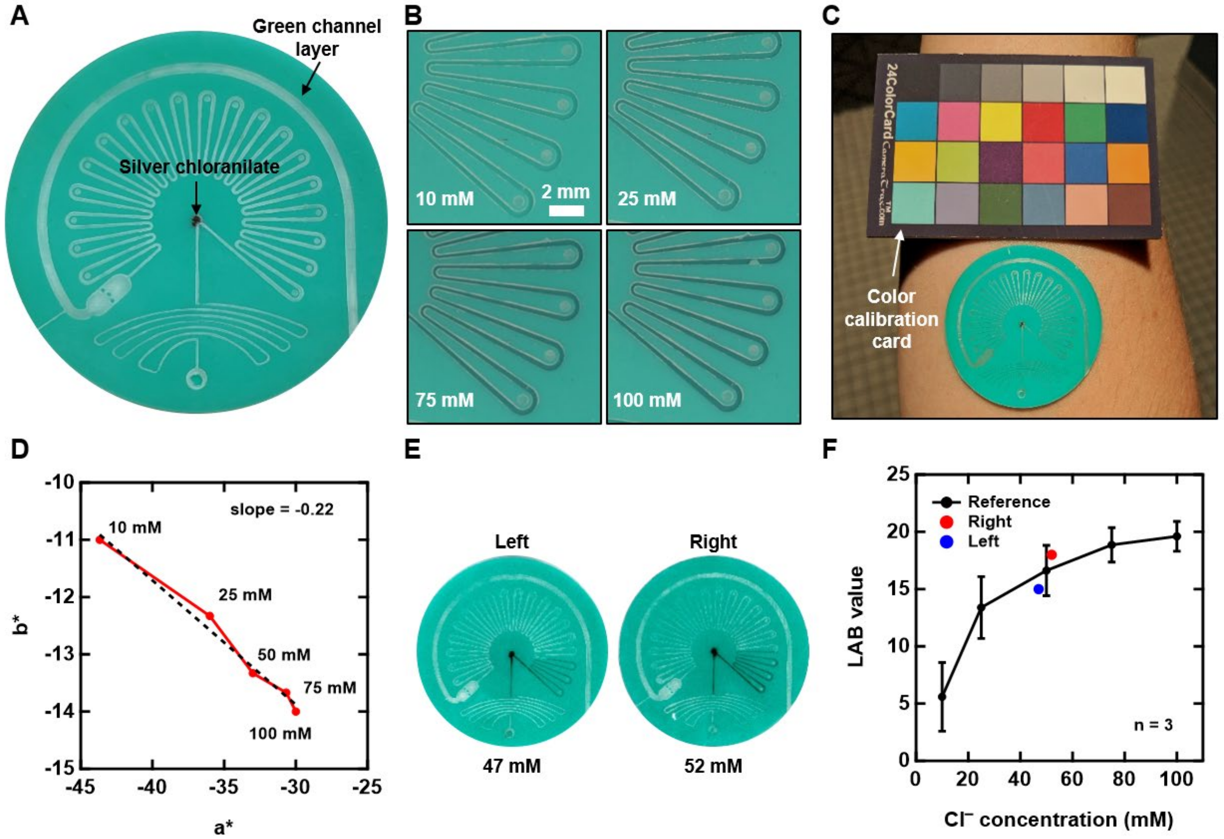

Supplementary Figure 4. **Chloride measurements.** A) A green molded layer provides a background for viewing both extent of fluid ingress via the microstructural optics as well as for colorimetric detection of sweat chloride via a reaction with silver chloranilate. Scale bar: 1 cm. B) Images of devices filled with reference chloride concentrations for calibration. C) A 24-panel color card is placed next to the device to enable color calibration in post-processing. D) a* and b* values for reference chloride measurements (LAB color space). Scale bar: 1 cm. D) Representative images of an on body chloride trial from the left and right volar forearms of a subject. F) Colorimetric detection of chloride sweat levels closely match those provided by a chloridometer. The LAB value represents the scaler projection of the measured color value onto the calibration curve in a* b* space as shown in panel D. The error bars indicate standard deviation.

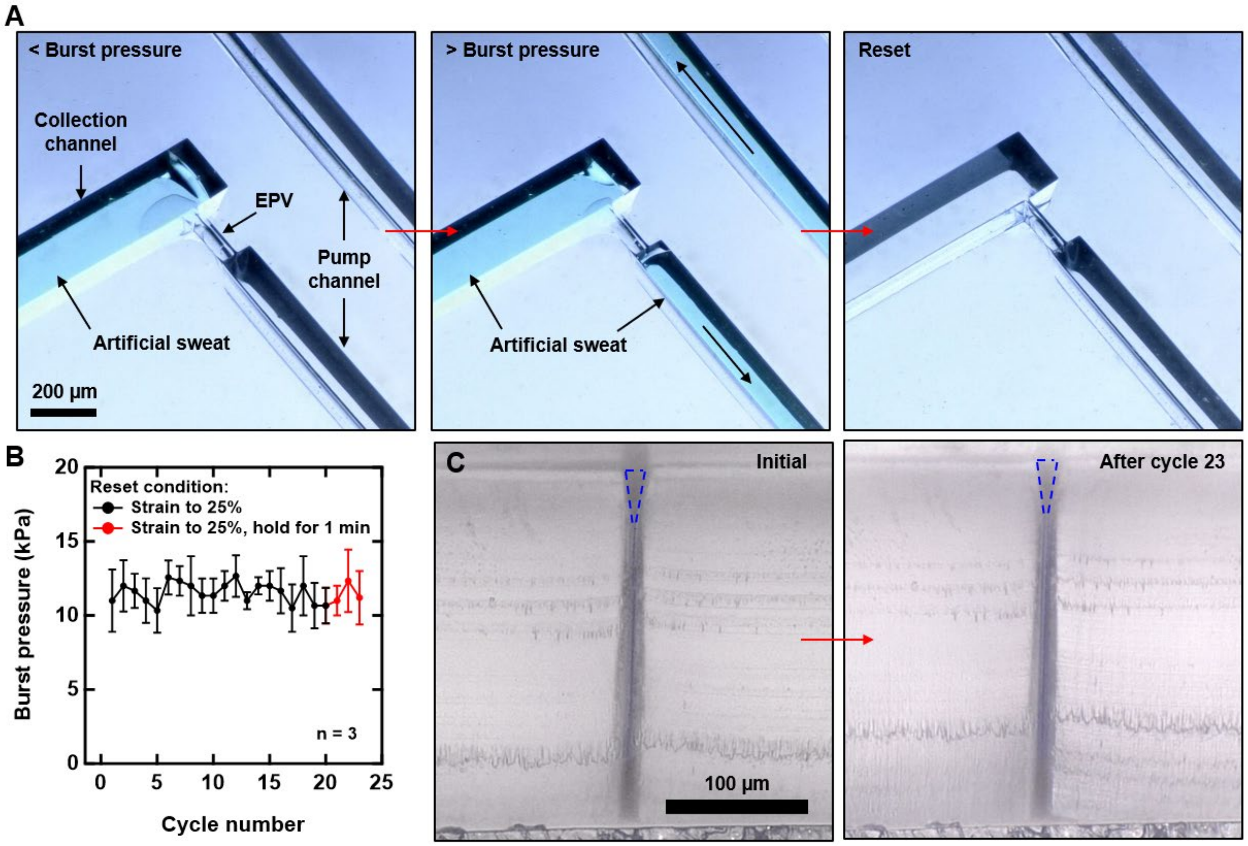

Supplementary Figure 5. **Elastomeric pinch valve repeatability.** A) Micrographs of an elastomeric pinch valve undergoing a burst pressure test. Before bursting (left), after bursting (center), and after resetting (right). No residual water droplets are observable due to the hydrophobicity of the PDMS. Scale car: 200 µm. B) No significant changes in burst pressure are observed for 20 cycles, or after an additional three cycles of 1 min hold. C) No significant changes in valve morphology are observed after cyclic burst pressure testing. Scale bar: 100 µm.

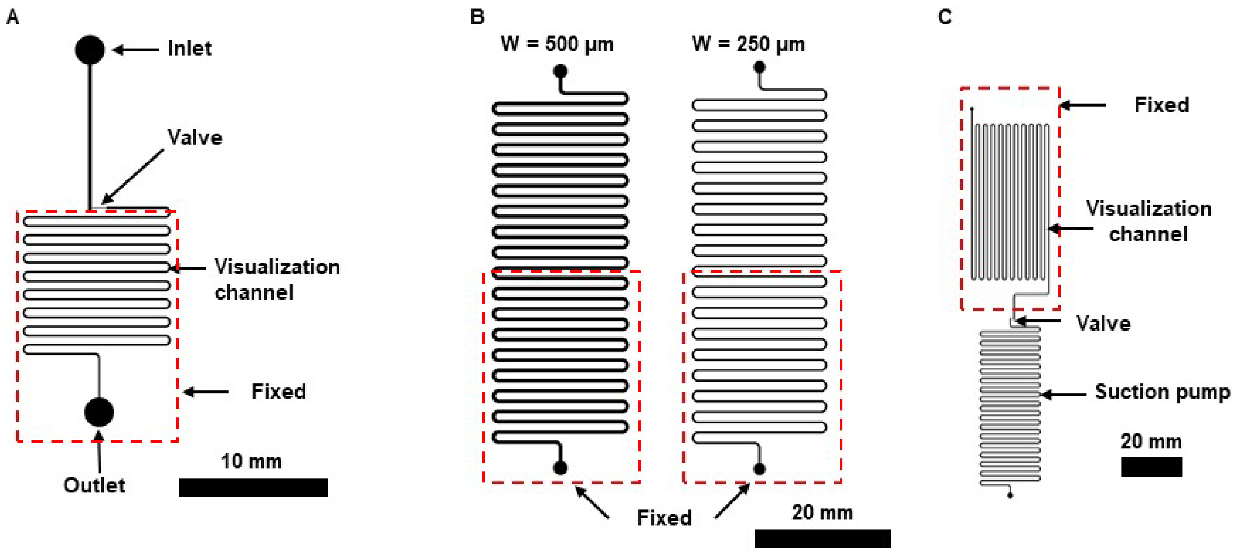

Supplementary Figure 6. **Structure geometries for systematic tests of the microfluidic components and systems.** A) Valve burst pressure tests. Scale bar: 10 mm. B) Pump tests. Scale bar: 20 mm. C) Purge tests. Scale bar: 20 mm.

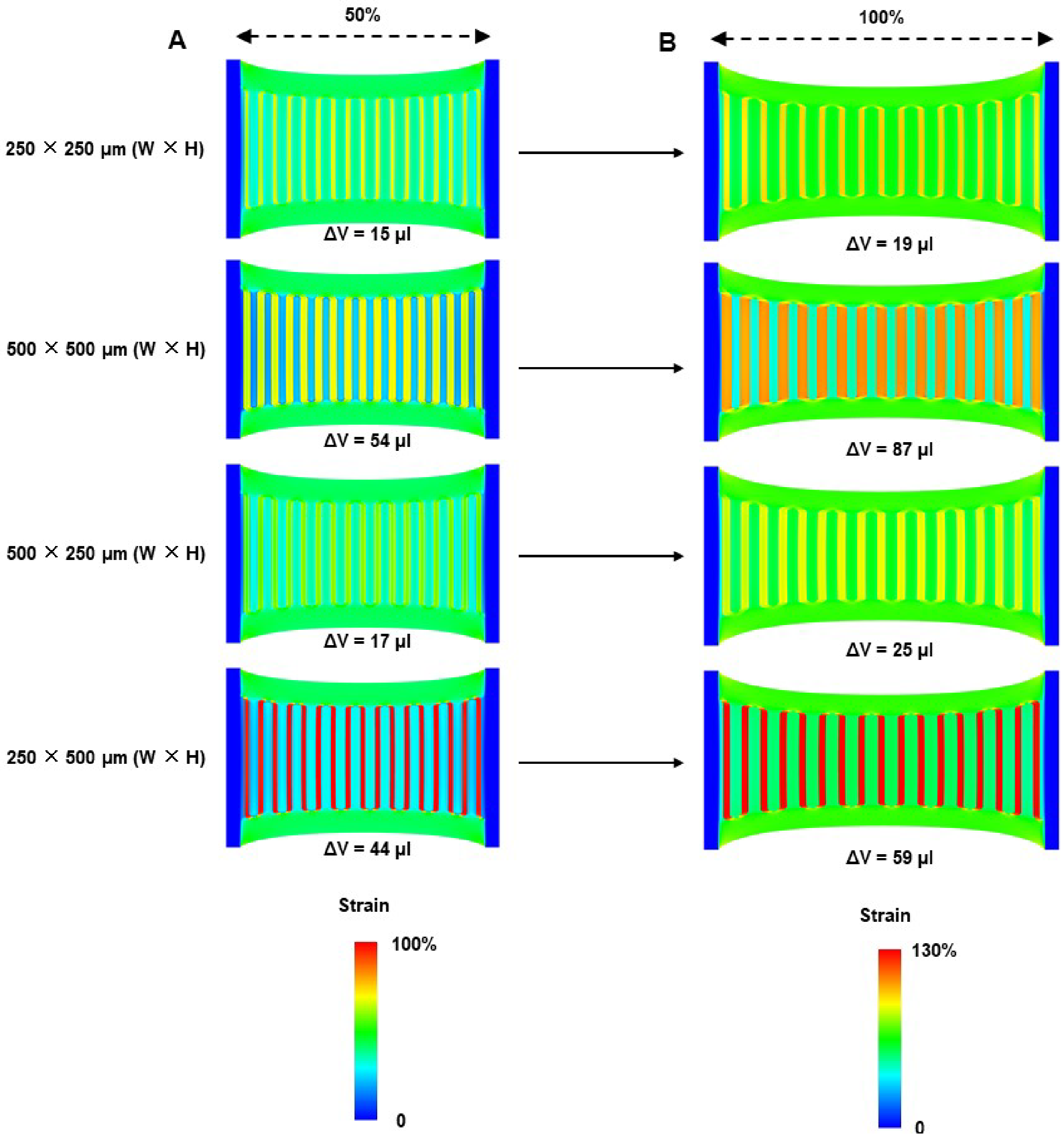

Supplementary Figure 7. **3D FEA of suction pump test structures**. A) 50% strain. B) 100% strain. ΔV = volume change.

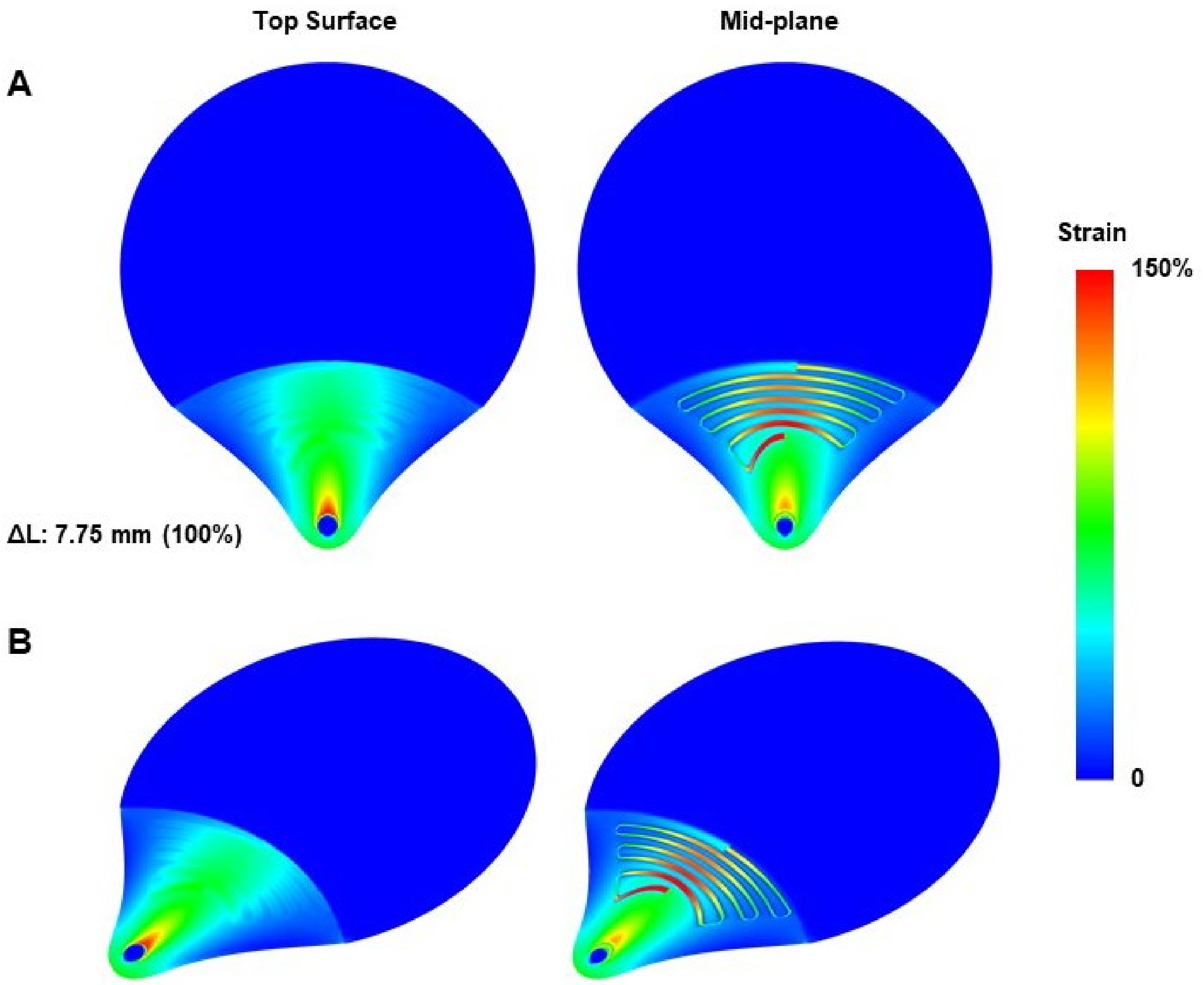

Supplementary Figure 8. **3D FEA of curved suction pump.** A) Top view. B) Oblique view. ΔL = displacement.

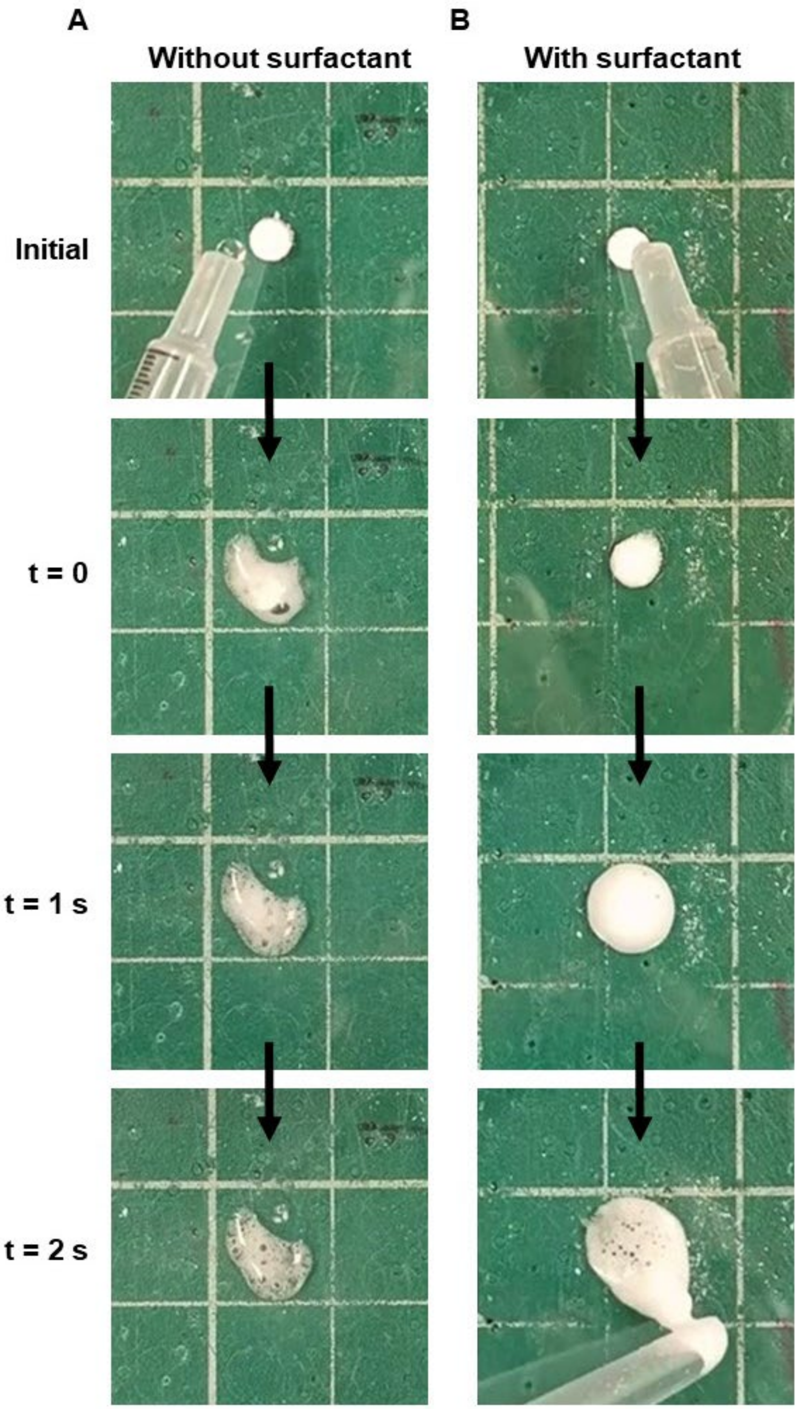

Supplementary Figure 9. Effect of surfactant on foaming behavior of the effervescent tablet. A) Images of a 1 mg tablet of sodium bicarbonate:citric acid (1.2:1 wt:wt) reacting with a droplet of water. B) Images of a 1 mg tablet of sodium bicarbonate:citric acid (1.2:1 wt:wt) with an additional 3wt% polysorbate 20 reacting with a droplet of water. Scale bar: 5 mm.

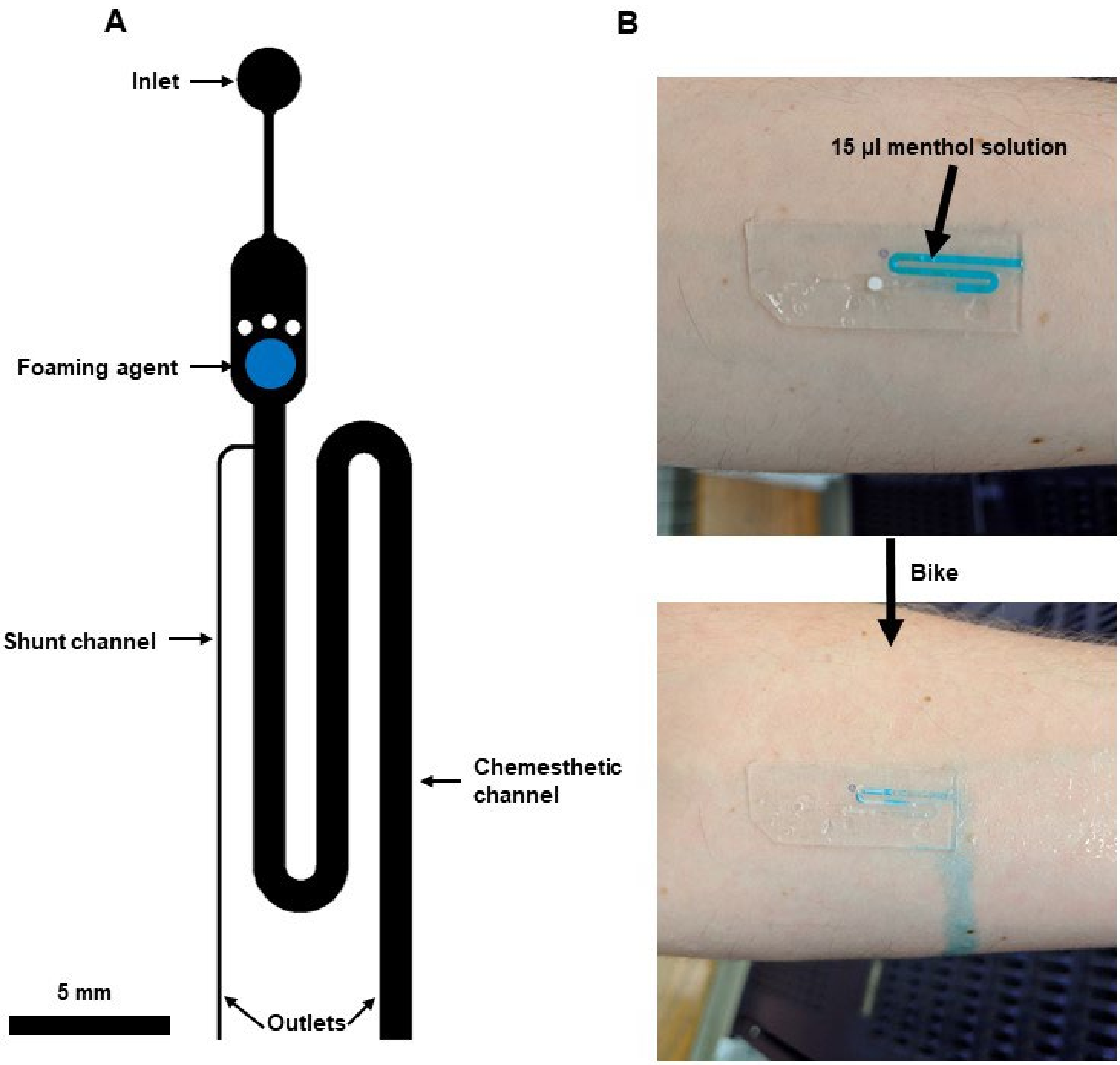

Supplementary Figure 10. **Chemesthetic trials.** A) Epifluidic test structures for on-body chemesthetic tests. Scale bar: 5 mm. B) Representative results for a chemesthetic trial of menthol. Scale bar: 10 mm.

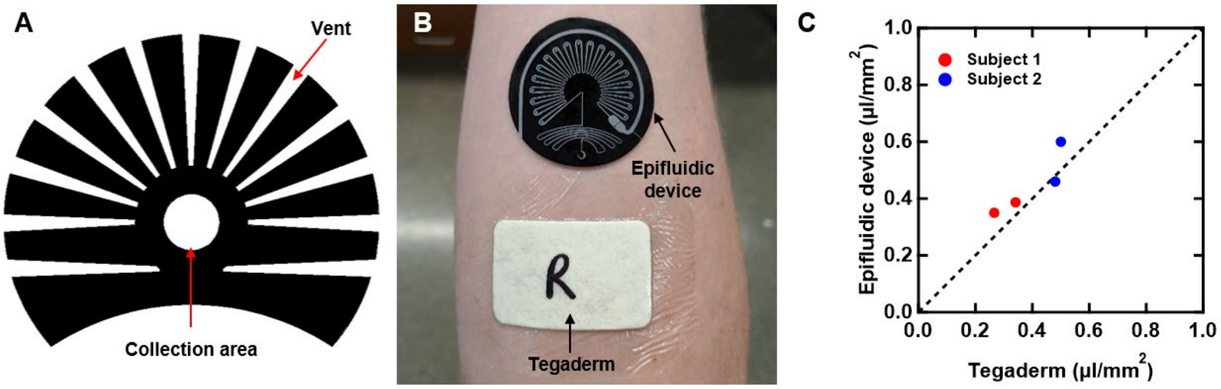

Supplementary Figure 11. Effect of patterned adhesive and compensatory sweating. A) Adhesive pattern including radial vents. B) An epifluidic device and Tegaderm absorbent pad mounted on the volar forearm. Scale bar: 2 cm. C) Areal sweat volume for epifluidic devices as compared to Tegaderm absorbent pads for two subjects.
